# Differential Private Deep Learning Models for Analyzing Breast Cancer Omics Data

**DOI:** 10.3389/fonc.2022.879607

**Published:** 2022-06-23

**Authors:** Md. Mohaiminul Islam, Noman Mohammed, Yang Wang, Pingzhao Hu

**Affiliations:** ^1^Department of Computer Science, University of Manitoba, Winnipeg, MB, Canada; ^2^Department of Biochemistry and Medical Genetics, University of Manitoba, Winnipeg, MB, Canada; ^3^Department of Electrical and Computer Engineering, University of Manitoba, Winnipeg, MB, Canada; ^4^Research Institute for Oncology and Hematology, CancerCare Manitoba, Winnipeg, MB, Canada

**Keywords:** deep learning, differential privacy, Rényi differential privacy, breast cancer, omics data

## Abstract

Proper analysis of high-dimensional human genomic data is necessary to increase human knowledge about fundamental biological questions such as disease associations and drug sensitivity. However, such data contain sensitive private information about individuals and can be used to identify an individual (i.e., privacy violation) uniquely. Therefore, raw genomic datasets cannot be publicly published or shared with researchers. The recent success of deep learning (DL) in diverse problems proved its suitability for analyzing the high volume of high-dimensional genomic data. Still, DL-based models leak information about the training samples. To overcome this challenge, we can incorporate differential privacy mechanisms into the DL analysis framework as differential privacy can protect individuals’ privacy. We proposed a differential privacy based DL framework to solve two biological problems: breast cancer status (BCS) and cancer type (CT) classification, and drug sensitivity prediction. To predict BCS and CT using genomic data, we built a differential private (DP) deep autoencoder (dpAE) using private gene expression datasets that performs low-dimensional data representation learning. We used dpAE features to build multiple DP binary classifiers to predict BCS and CT in any individual. To predict drug sensitivity, we used the Genomics of Drug Sensitivity in Cancer (GDSC) dataset. We extracted GDSC’s dpAE features to build our DP drug sensitivity prediction model for 265 drugs. Evaluation of our proposed DP framework shows that it achieves improved prediction performance in predicting BCS, CT, and drug sensitivity than the previously published DP work.

## 1 Introduction

In drug discovery research, one of the crucial steps is to test the drug’s sensitivity (i.e., the drug’s effectiveness to inhibit a particular biological function). Machine learning (ML) models can predict such a drug response in cell lines using gene expression data instead of time-consuming and expensive wet-lab experiments (1). Min et al. (2) proposed a deep learning (DL) based framework (DeepDSC) to predict drug sensitivity using GDSC dataset (3). They achieved improved prediction performance than the baselines in terms of root-mean-square error (RMSE) and coefficient of determination. Ahmed et al. (4) used graph based DL approach which was evaluated on the GDSC and showed improved prediction performance than the shallow models e.g., Random Forest and Support vector machines. Recently, Shuangxia et al. (5) proposed another DL based framework (DeepGRMF) to predict drug sensitivity. DeepGRMF was evaluated using GDSC and showed superiority than the baselines. DeepGRMF used DL, graph model and matrix-factorization to collect various set of drug chemical structures for the final prediction of the response of a drug to single cell-lines.

However, a data custodian may not want to publicly release a genomics dataset to build an ML model because of the privacy-sensitive nature of gene expression data (6). We know that an exposed genome data can be used to know everything about a person, such as the possibility of misery from a disease and life expectancy (7). Therefore, access to private sensitive genomics data often goes to the applicants after an application process with a nondisclosure agreement and a thorough background check. This process limits data availability to a broader audience, which negatively affects the development speed of biological insights for various problems (e.g., risk gene identification of diseases). Therefore, we need to develop a framework that will promptly acquire a sensitive genomic dataset and perform problem-specific analysis without divulging the individuals’ private information in the dataset.

Recently, for the first time, Honkela et al. (8) used the differential privacy (DP) mechanism in drug sensitivity prediction. DP is a rigorous privacy incorporation approach that permits researchers to access and analyze genomic data while provides a mathematical guarantee of individuals (i.e., participants in the study) privacy (9). According to Dwork et al. (10), a randomized algorithm (*AL*) is called ∈-DP if *AL* can produce output (*OUT*) for the two neighboring datasets *DS* and *DS*^′^ (i.e., differed by at most one data record) which holds the equation 1.


(1) $$Prob\left(Al\left(DS\right)=Out\right)\leq e^{\in }Prob(Al(DS^{\prime })\;=\;Out)$$

In general, the DP preserves an individual’s privacy by injecting random Laplacian noise into the published statistical outcomes that were processed from sensitive personal information. Intuitively, the random noise is brought into the data in a way that the statistical outputs (e.g., disease status) from the raw and noisy datasets are similar up to a factor (exp ∈). In this way, every patient who participates in the study achieves plausible deniability about a specific outcome. Hence, we can say that if a model is ∈-differential private, then an adversary who knows every patient’s private information in the dataset except for one single patient, can not infer with high confidence (depends on ∈), about that unknown patient’s private information. Privacy budget (∈) refers to the maximum amount of private information a DP model can leak. A smaller value of ∈ corresponds to tighter privacy protection. From the ∈ = 1.0-DP model, an adversary can not be more certain about a participant’s outcome than to the multiplicative factor of *e* ^∈ = 1.0^ = 2.718 compared to the actual outcome of that participant in the study. However, Honkela et al. (8) approach for drug sensitivity prediction is suffering from the high-dimensionality nature of gene expression data.

We need to produce useful results from sensitive genomic data analysis without violating individuals’ privacy. DL has become the most effective ML approach to process genomic data in recent times. A DL model can analyze high dimensional data (e.g., gene expression) (11) and achieves better prediction performances while keeping the privacy of the data intact (12). Besides, DL has the ability of automatic trainable feature extraction from high-dimensional data to achive state-of-the-art predictions, such as image classification (13). Of note, if we train a non-private DL model with the sensitive data, it becomes vulnerable to privacy inference attack (14) and model inversion attack (15).

Breast cancer is a common and fatal disease, and it appears that normal tissue is converted to tumor pathology. A usual and successful means of detecting this disease are mammogram images. Previously, DL based methods have shown promises to extract fine-details from image data for further classification of an image. Therefore, Altan (16) proposed a convolutional neural network (CNN) based DL framework to classify Mammograms as cancer-normal. Then, Altan (17) extracted only the region of interest from the Mammograms (ROIs) to apply CNN and deep autoencoder based architecture to separate cancer-normal patients. In both cases, DL based approaches were able to achieve high prediction performance for classifying patients to cancer-normal in terms of accuracy, sensitivity, specificity and precision. Then, Altan (18) uses Deep belief Networks to classify ROIs. This framework also achieved similar prediction performance compared to (16, 17)

In addition, an obvious limitation of the DL approach is that it requires lots of training examples to optimize a massive number of parameters. In real-life scenarios, one source of sensitive data (i.e., genomic data) may not always have much labeled data. To overcome this limitation, collaboration among the genomic data custodians is necessary. In addition, genomic data sharing among many researchers leads to the development of new biological insights (19). Nevertheless, the collection of large volumes of genomic data may violate individuals’ private data (20). We can do such collaboration while keeping the privacy of the data from multiple data custodians by leveraging one of DL’s attractive properties, i.e., transfer learning. Transfer learning allows us to transfer the knowledge learned by a model for one task to another second task model.

We know that genomic data contains both categorical (e.g., disease status) and continuous data (e.g., expression levels of genes). Thus, we can use genomic data to build regression models for different regression tasks (i.e., logistic regression and linear regression). Unfortunately, an adversary can infer an individual’s participation in the study by analyzing the regression coefficients of a published regression model (21).

Chaudhuri et al. (22) introduce a ∈-differential privacy solution for the differentiable and convex objective functions of a logistic regression task. We can not use this approach in practice because most of the real-world regression problems follow non-convex regression objective functions. Hence, the authors modify the input to achieve a convex regression objective function. Besides, Kifer et al. (23) extended this approach for a convex objective function based linear regression task. To overcome the compulsory requirement of a convex objective function, Zhang et al. (24) introduced a new approach called Functional Mechanism (FM) to adapt ∈-differential privacy to both types of regression tasks. FM can ensure ∈-differential privacy for non-convex standard regression problems even when the output space is unbounded.

Niinimäki et al. (25) overcame the limitation of Honkela et al. (8) by using the transfer learning in a differential privacy framework, which achieved state-of-the-art prediction performance (∈ = 10) using gene expression-based genomic datasets. They collected the TCGA and the GDSC datasets as a public and private datasets respectively. They also redistributed the TCGA dataset to match the data distribution of the GDSC. Later, they built a non-private data representation learning model (variational autoencoder (26)) with the public dataset. This model was used to extract a new representation (i.e., transfer learning) of their private data. These newly represented data were used to build DP based classifier (22) to classify cancer types from the TCGA dataset, and DP based linear regression models (23) to predict drug sensitivity from the GDSC dataset.

The primary limitation of Niinimäki et al. (25) is that there must be a publicly available dataset to train a data representation learning model. However, requiring a public dataset is not practical for real-life scenarios. We may not always find a publicly available dataset that is similar to a private dataset. It is possible to find a similar dataset from a private source. Besides, the amount of noise Niinimäki et al. (25) insert into the data depends on the cardinality. Hence, this framework (25) can be used for a small training set only. Niinimäki et al. (25) also requires the redistribution of the public dataset according to the private dataset. Intuitively, a public dataset redistribution according to a private dataset is an apparent privacy violation.

Differential privacy has several attractive properties, such as composability, i.e., if all model components are differentially private, then the model becomes differentially private. For example, if a DL model with two components (i.e., different batches of training data) with a privacy budget ∈_1_ and ∈_2_ has access to a private dataset, the complete DL model can achieve differential privacy with a privacy budget ∈_1_ + ∈_2_ . Besides, DP based models are invariant to post-processing, such as model inversion attack (14, 15). Hence, Shokri et al. (27) first introduced differential private DL model.

Abadi et al. (12) showed that the ∈-differentially private DL models suffer from the low utility in several applications. As a result, they used a relaxed version of differential privacy (equation 2) called (*ϵ*,*δ*)-differential privacy (28) to build their differentially private DL models. A DL model is (∈, *δ*)-differentially private if it achieves ∈-differential privacy with a high probability *δ*. This form of relaxing differential privacy is useful for a complex optimization problem when a stricter version produces useless results (29). However, a(∈, *δ*)-DP model achieves (∈)-DP with probability (1-*δ*). In (12), authors introduced Gaussian noise into the trainable parameters’ gradients. They inserted noise during the stochastic gradient descent computation of the training phase and achieved ∼ 90% accuracy on the MNIST data set at ∈ = 0.5 (∈ = privacy budget and lower ∈ signifies tighter privacy in the model). However, Mironov (30) introduced Rényi differential privacy (RDP) which overcomes the information leaking problem of amount (1-*δ*) by (∈, *δ*)-differential privacy. Recently, Triastcyn and Faltings (31) introduced a Bayesian differential privacy (Bayesian DP) mechanism, which focuses on the dataset specific data distribution. However, in the worstcase scenario, Bayesian DP may fail to protect the training data from an adversary.


(2) $$Prob\left(Al\left(DS\right)=Out\right)\leq e^{\in }Prob(Al(DS^{\prime })=Out)+\delta$$

Phan et al. (32) was the first work to build a DP-DNN autoencoder that can provide state-of-the-art regression performances such as the prediction of human behaviors from health social networks. They used FM((24)) to perturb the objective function’s coefficients to build DP-DNN. However, FM((24)) follows ∈-DP which may affect the performance of Phan et al. (32) framework in many real life applications (29).

In this study, our goal is to build a framework to perform three specific tasks: first, build a (*α*,*∈*)- Rényi differential private (RDP) DL based data representation learning model (dpAE) from a private dataset; second, transfer the learned knowledge from dpAE to build a (*α*,*∈*)-RDP DL based binary classifiers; third, transfer knowledge from dpAE to build (*α*,*∈*)-RDP DL based linear drug sensitivity regressors while producing improved utility then the related previously published approaches (8, 25, 32). We consider anyone as an adversary who wants to identify *i^th^* particular participant in the dataset.

We assumed there are two data sources. The first data source has a private dataset (PD_1_) with a small number of samples. The second data source has another private dataset (PD_2_), with a larger number of samples with the same set of features as the PD_1_. Then, we built a DP based autoencoder (dpAE) using PD_2_. We used dpAE as a data representation learning model as well as a data dimensionality reduction technique. Afterward, we used this dpAE to map (i.e., transfer learning) the data from PD_1_ into a lower dimension space. Finally, we used these lower-dimensional DP features of PD_1_ to build DP based DL models: dpClassM (to predict cancer type or cancer status of a breast cancer patient) and dpRegM (to predict drug sensitivity). Of note, the components in our proposed model are differential private. Hence, according to DP’s composability property, the final models (dpAE, dpClassM, and dpRegM) are also deferential private.

Experimental evaluation indicates that the proposed framework achieves improved prediction accuracy (i.e., utility) in DP cancer type (CT) and breast cancer status (BCS) prediction than the baseline works (25, 32). We also have improved Spearman’s rank correlation coefficient while ensuring better privacy in DP drug sensitivity prediction than the previously published state-of-the-art approachs (8, 25). Therefore, according to the experimental results, one can use our proposed framework to integrate multiple private datasets to build robust DL models while providing a robust privacy guarantee for the privacy-sensitive raw input data.

This paper is organized as follows: Section 2 describes our proposed differential private DL framework for BCS, CT, and drug sensitivity prediction and the datasets that were used during the experiments, then follows Section 3 that presents and discusses our experimental findings, and finally, Section 4 presents our conclusions.

## 2 Materials and Methods

### 2.1 Datasets

Contemporary large-scale pharmacogenomics research e.g., the TCGA (33) and GDSC (3) provides valuable information to computational drug discovery such as prediction of cell-drug response (GDSC) orcancer outcome (TCGA). METABRIC (34) dataset can be used to predict estrogen receptor +/- using privacy sensitive copy number variations. In this study, we have collected datasets to build privacy incorporated deep learning frameworks. All these datasets are publicly available. However, for the experimental purposes, we have collected and treated them as private sensitive data to mimic private datasets (i.e., contain sensitive private information) concepts.

First, we collected the Genomics of Drug Sensitivity in Cancer (GDSC) project (3) data. We pre-processed GDSC similarly to the previous work (25). After the pre-processing, the GDSC has 985 cell lines, and each of them has microarray-based gene expression data of 11,714 genes. Besides, the GDSC dataset has the half-maximal inhibitory concentration (IC50) of 265 drugs (i.e., drug sensitivity) in cancer cell lines. A lower IC50 means higher sensitivity of the drug on the cell line. Second, we collected a privacy-sensitive dataset called METABRIC (Molecular Taxonomy of Breast Cancer International Consortium) (34) in order to perform experiments for breast cancer subtypes (estrogen-receptor-positive (ER+) or estrogen-receptor-negative (ER-)) classification. METABRIC contains copy number alteration (CNA) data for each patient. Such CNA data is a type of Copy number variation (CNV) data representing the copy number gain or loss or diploid information of DNA fragments (i.e., genes) in the genome. An adversary can use such exposed CNV data to know about a specific genetic disorder or complex diseases such as autism, cancer, immune deficiency, and neurodegenerative and neuropsychiatric disorders (35). Hence, it is essential to have a privacy mechanism in the CNV type data analysis pipeline to protect individuals’ privacy from the adversary. In METABRIC (34), we have three discrete copy number calls for each gene of a patient (18,000 genes/patient): −1= copy number loss, 0= diploid, 1= copy number gain in our CNA mutation matrix (patients-by-genes). Of note, we have 991 samples (794 samples for ER+ and 197 samples for ER-) and 984 samples to train and test a binary classifier to predict ER status (ER+-), respectively. Third, similar to (25), we collected pre-processed version of TCGA from https://xenabrowser.net/datapages/. We assembled the preprocessed version of the pan-cancer RNA-seq gene expression data from the TCGA while removing low expression genes. Then, 14,796 genes were left to represent each of the 10,534 patients from 33 different cancer types.

Before proceeding further, we unified TCGA and GDSC datasets together in the framework by the genes from the TCGA and GDSC datasets which are present in both these datasets. Similarly, we unified TCGA with METABRIC and METABRIC with GDSC

### 2.2 Methods

**Figure 1:**
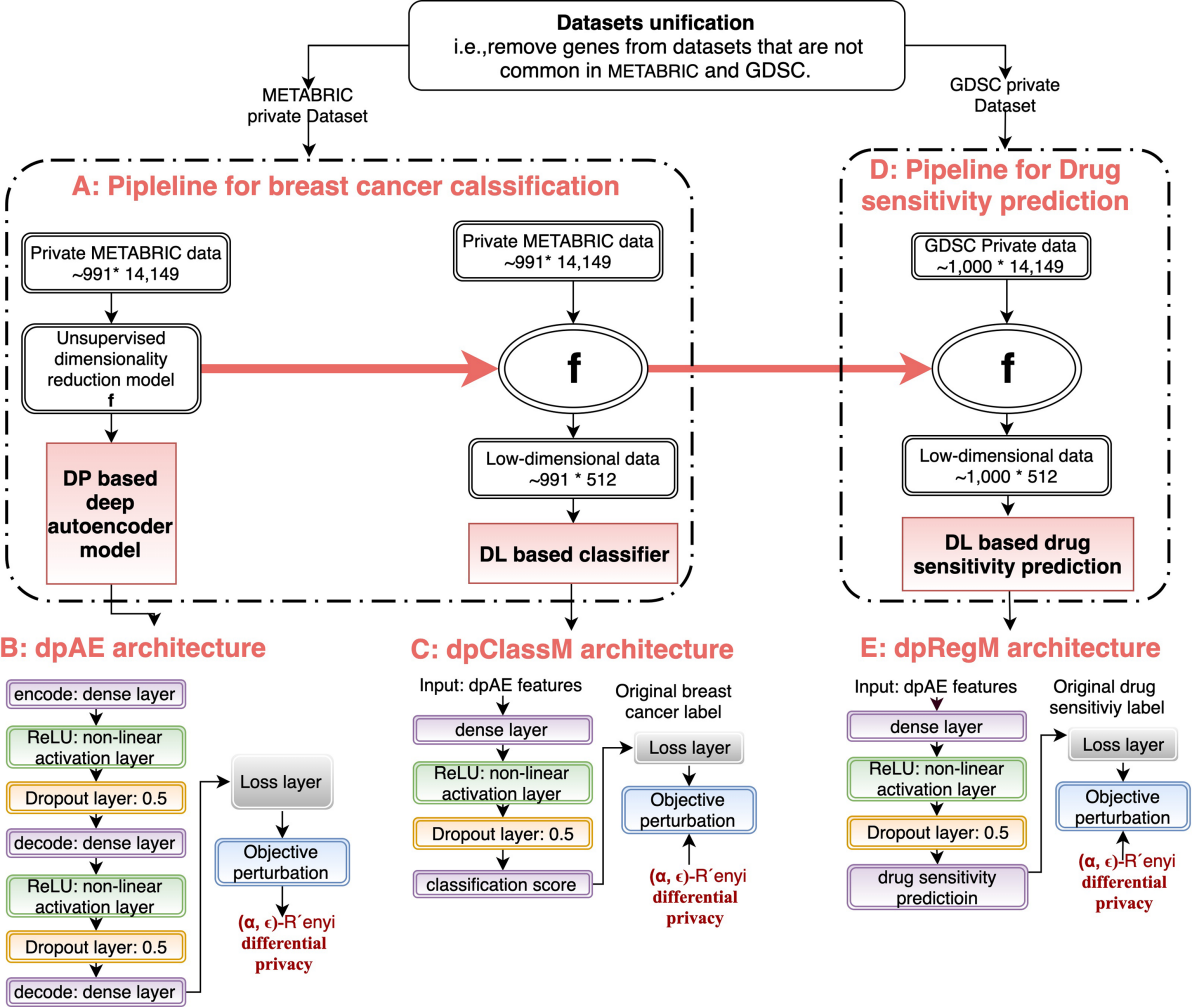
Proposed deep learning based differential private framework to perform classification and linear regression tasks with privacy-sensitive biological data. **(A)** Pipeline to build an underlying data representation learning model (i.e., dpAE) with private data. **(B)** DL architecture of dpAE. **(C)** DL architecture of our proposed differential private classifier (i.e., dpClassM). **(D)** Pipeline to predict the sensitivity of drugs in cell lines. **(E)** DL architecture of our proposed differential private linear drug sensitivity regressor (i.e., dpRegM).

In this study, we proposed a framework to predict patient cancer outcome, and sensitivity of drugs in a cell line while preserving every patient’s private information (Algorithm 1) in the datasets. **Figure 1** shows the details pipeline of our proposed framework.

#### 2.2.1 Rényi Differential Privacy

We used Rényi differential privacy (RDP) (30) definition to make our DL models *∈*-differential private. RDP is a natural relaxation form of *∈*-DP (10) while overcomes the limitation of relaxed (*ϵ*,*δ*)-DP (28). Unlike *∈*-DP (10) the RDP, which inserts Gaussian noise to the model parameters, we can use it for training a DL model by leveraging the property ‘closed under the addition’ of Gaussian noise. According to RDP, if a DL model is ∈-DP then, all the batches during the training are also *∈*-DP (composition property). However, RDP used Rényi divergence to produces a random variable under a constraint (*α*) instead of the multiplicative factor *e^∈^*.

Definition (Gaussian mechanism): If the randomized algorithm *Al* for a dataset *DS* produces *N*(*Q*(*DS*),*σ*^2^*Ik*) then for the neighboring datasets *DS* and *DS*^′^, and *α* = (1,∞), Gaussian mechanism can be defined as RDalpha $\left(\text{A}1\left(\text{DS}\right)\right)\;\|\text{A}1\left({\text{DS}}_{2}\right)\leq \frac{\alpha \Delta_{2}^{2}\left(Q\right)}{2\sigma^{2}}$ . Mirnov (30), provides mathematical guarantee that *Al* achieves (*α,∈*) -RDP when $\sigma^{2}=\frac{\alpha \Delta_{2}^{2}\left(Q\right)}{2\in }$ .

Here, *Q* is the vector-valued function for the queries in the dataset. Hence, this function represents the sampling rate for each of the samples in the dataset. We know the main component to ensure RDP is the addition of Gaussian noise. Such noise is dependent on the *ℓ*_2_ sensitivity of *Q*. Therefore, we insert the appropriately scaled noise to perturb the learning weights based on Q. Besides, *Δ*_2_ =*ℓ*_2_ sensitivity of Q = max ‖*Q*(*DS*)−*Q*(*DS*')‖_2_ where *DS* and *DS*^′^ datasets are same except one record.

Definition (Composition property): The composition property of RDP allows us to apply Rényi differential privacy in a DL model as we train our models using batch-wise training fashion. This property states that if two randomized algorithms A_1_ and A_2_ for two different data batches of samples have parameters (*α*, *∈*_1_) – and (*α,∈*_2_), which are Rényi differentially private respectively, then the randomized algorithm defined as (A, B), where A∼A_1_ and B∼A_2_ (A), satisfies (*α*, *∈*_1_+*∈*_2_)-RDP. RDP uses an accountant function to keep track of the privacy parameter that gets spent for each batch training.

**Algorithm 1** Differential private deep learning based classification and linear regression framework.

**input:** D_M_= The private METABRIC dataset,

_G_ = The private GDSC dataset,

label_class_ = Breast cancer status i.e., ER+/- labels of D_M_

label*_drugSensitivity_* = Drug sensitivity of D_G_

Here, *H* = Hyperparameters, *I* = Indicator, *F* = Features, *L* = Labels, *P* = Performance

**output:** Public release of the dpAE, dpClassM, and dpRegM models.

**initialization:**

dpAE_H_ = {number of layers, learning rate, training epochs}, P = [0,0], Indicator = 10

**Pseudocode:**

**While** *I* > 0

Build dpAE:*f*(D*_M_*) → Low-dimensional D*_M_*

dpAE*_F_* ← dpAE(D*_M_*)

Build dpClassM:*f*(dpAE*_F_*, *label_class_*) → ER+/-‖

*dpClassM_P_* ← [accuracy(dpClassM), AUC(dpClass)]

**if** dpClassM*_P_* ≥ *P* **then**

P ← dpClassM*_P_*

dpAE*_H_* ← update with new settings

I ← 10

**else**

dpAE*_H_* ← update with new settings

I ← I - 1

**end if**

**end while**

Publish dpAE

**if** Breast cancer status classification **then**

Publish dpClassM

**end if**

**if** Drug sensitivity prediction **then**

dpAE*_F_* ← dpAE(D_G_)

Build dpRegM: *f*(dpAE*_F_*, label*_drugSensitivity_*) → drug sensitivity

Publish dpRegM

**end if**

#### 2.2.2 Differential Private Deep Autoencoder

We followed the idea of the stacked denoising autoencoder (SDAE) (36) to build an underlying data representation learning model. Unlike the traditional autoencoders, SDAE can predict a robust lower dimensional output representation of the input even if the input data is corrupted. This is helpful in our case, as we are building this autoencoder to predict the low-dimensional equivalent output from another dataset with different data distribution. Previously published independent researchers have showed that we can insert random noise into the gene expression input data during the training process. Then, denoising autoencoder is capable of extracting robust stable biological principles between genes from genome-wide expression data (37, 38). In our framework, we inserted noise from random normal distribution with mean 0 and a range of standard deviations [0.1, 0.2,.3]. We achieved the optimal prediction performance for standard deviation 0.1. The higher-level architecture of dpAE is shown in **Figure 1B**. We used dpAE as a lower-dimensional data representation learning model. In the beginning, we inserted a random noise into the raw original input (ROI). Then, we pass this noisy input to the encoder. We encoded the input data with three fully-connected (dense) layers of 8000, 4000, and 2000 sizes. Each neuron of a dense layer receives input from all of the previous layer’s neurons. A dense layer performs a regular matrix multiplication and passes the output to the next layer. Next, we transformed each layer’s output using the rectified linear unit (ReLU) to introduce non-linearity into our dpAE model. A ReLU layer converts all the negative values into zeros. We used a Dropout layer (39) after each ReLU layer to improve the model’s performance over the unseen data. The dropout layer randomly drops some neural, forcing the network to learn general weights for each neuron. Then, we pass this encoder’s output to the decoder. Here, our objective is the reconstruction of the ROI. This decoder also consists of three dense layers of sizes 4000, 8000, and the original input dimension. Each of these layers tries to reproduce the output from the encoder’s associated same size layers. Similar to the encoder, a ReLU and a dropout layer follow these dense layers. Then, we used a loss layer, which acts as the objective function to calculate the loss between the reconstructed input and the ROI. This loss represents the similarity between the reconstructed input and the ROI (lower loss represents a higher similarity).

To make dpAE (*α*,*∈*)-Rényi differential private (i.e., (*α*,*∈*)-RDP), we inserted Gaussian noise into the reconstructed loss gradients. Then, we used these noisy gradients to update the model’s trainable weight parameters. Next, we used these perturbed model parameters to minimize the reconstruction error. Consequently, according to the DP’s composability property, the complete dpAE model is also *α*,*∈*-Rényi differential private.

#### 2.2.3 Differential Private Deep Learning Based Classification Model

In this study, we proposed a framework (**Figures 1A–C**) to build a (*α*,*∈*)-RDP DL model (i.e. dpClassM) to predict ER+ or ER- using gene expression data.

We used the dpAE to extract the lower dimensional differential private representation for the METABRIC train dataset, i.e., dpAE features. These dpAE features were processed by a dense, ReLU and dropout layer. The processed dpAE features were used as input to a 2-size dense layer to get the final prediction scores for ER+ and ER- classes. We converted these prediction scores into prediction probability using a softmax (40) function. We used the cross-entropy loss function to calculate the error between prediction and ground truth of the input. We stopped the training of dpClassM when there is no improvement in the model performance for ten consecutive times. In Algorithm 1, the variable ‘Indicator’ is used to perform the stopping of dpClassM training.

We followed (30) approach to introduce Gaussian noise into the gradients (which were calculated with respect to the model parameters’) of the objective function to make dpClassM the (*α*,*∈*)-RDP. Finally, we used a stochastic gradient descent approach to train dpClassM in batches of training samples with these perturbed model parameters. Let us assume we have ten batches of samples to build dpClassM, and outputs from each of these batches are (*α*,*∈*)-RDP. Then according to the composability property of DP, dpClassM is (*α*, *∈*_1_ + *∈*_2…… +_ *∈*_10_) -differential private. Intuitively, the complete dpClassM model leaks ten times more private information than the given privacy budget *ilon*. This is undesirable, which led us to use an accountant function (similar to (12)) to distribute given *∈* into each batch so that the privacy budget of dpClassM do not exceed *∈*.

#### 2.2.4 Differential Private Deep Learning Based Linear Regression Model

**Figure 1** shows the proposed framework to build (*α*, *∈*)-RDP DL model (i.e., dpRegM) to predict the sensitivity of drugs in cancer cell lines. At first, we built a (*α*, *∈*)-RDP based dpAE using the private METABIRC dataset. We used a trained dpAE to extract low-dimensional (*α*, *∈*)-RDP representations (i.e., dpAE features) of our private GDSC dataset. This new private lower representation of the GDSC was used as input into the architecture of dpRegM (**Figure 1E**). We then transform the DP representation using a set of dense layers, insertion of non-linearity (ReLU layer), and a dropout layer. The last dense layer produces only one output, which we treated as the predicted sensitivity of drugs in *α*, *∈*-RDP cell line data. Similar to the building of dpClassM strategy, we used Gaussian noise-based mechanism of Mironov (30) to make dpRegM (*α*, *∈*)-RDP. Then, we trained the dpRegM using a standard batch-wise stochastic gradient descent approach.

## 3 Results and Discussion

In this study, we performed our experiments using the TensorFlow software (41) to build differential private BCS (i.e., ER+ or ER-) and CT classifiers, and drug sensitivity regressor. We have considered GDSC and TCGA as private datasets, while the METABRIC is an actual private dataset. In all the experiments, baseline Bayesian DP (31) uses high-dimensional original raw data as input to solve binary classification and linear regression tasks.

### 3.1 Differential Private Classifiers

**Figure 2:**
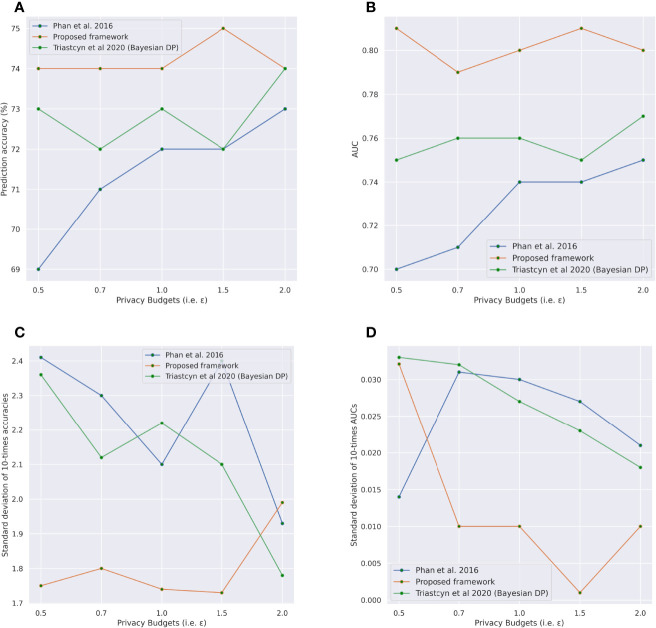
Comparison of prediction performances between our proposed Rényi differential private binary (ER+/-) classifiers and baseline models for different privacy budgets on the METABRIC (34) dataset. **(A, B)** Comparison of ER+/- classifiers in terms of mean accuracy (%) and mean AUC, respectively, from the 10-times repeated experiments **(C, D)** Comparison of standard deviations of accuracy (%) and AUC, respectively, from the 10-times repeated experiments.

We used the METABIRC data to build our dpAE and dpClassM binary classifiers to classify patients, either ER+ or Er-. We used two popular metrics, accuracy and Receiver Operating Characteristics (ROC) Area Under the Curve (AUC), to measure our classifiers’ effectiveness. **Figures 2A, B** shows our proposed dpClassM’s ER+/- prediction performance for the METABRIC test dataset. We have the best prediction result, 76% accuracy (**Figure 2A**), and 0.78 AUC (**Figure 2B**) when *∈* = 20 (we repeated the experiment for 10 times and reported the mean accuracy and AUC with standard deviation in **Figures 2C, D**). This figure also compares our predictions with the baselines (31, 32) prediction performances. However, **Figures 2A, B** shows that our proposed approach for the METABRIC dataset (for ER+/- classification) achieved improved accuracy and AUC for each of the predefined *∈*s than the baseline. However, we can not use Niinimäki et al. (25) approach for the METABRIC dataset as their approach requires a public dataset for representation learning, and METABRIC contains real private data.

In addition, we also performed experiments on the TCGA dataset to build a dpAE and cancer type classifier. **Supplementary Figure S1** shows the pipeline for the drug sensitivity prediction when the TCGA dataset is used to build a low-dimensional data representation learning model. The TCGA dataset contains a patient’s outcome for 33 distinct cancer types. We need to build $\left({}_{2}^{33}\right)$ binary classifiers to predict patient’s cancer types in order to cover all possible combinations of cancer type pairs (i.e., two cancer types at a time from the 33 available cancer types) in the TCGA. The supervised binary classification task is relatively easy for some of these cancer types pairs. Therefore, Niinimäki et al. (25) used a non-differential private classification approach to rank all the $\left({}_{2}^{33}\right)$ pairs of cancer types based on their difficulty of prediction in a binary classification setting. **Supplementary Table S1** shows the top 16 pairs of cancer types, which are difficult to predict. Among these pairs of cancer types, we choose to perform our experiments for the eight numbered cases in **Supplementary Table S1** to facilitate a direct comparison of our experimental outcomes with the baseline (25).

The first step of the proposed cancer type classifier framework (**Supplementary Figures S1A–C**) is to split the TCGA dataset. In this case, our first private dataset (PD_1_) has data for one of the cases from **Supplementary Table S1**, and patients for the remaining 15 pairs of cancer types go to the second private dataset (PD_2_). We used the PD_2_ dataset to build our (*α*, *∈*)-RDP data representation learning model (dpAE). Then, we used dpAE to extract 2000-size low-dimensional representations for each of the PD_1_ patients. These dpAE representations were used to build (*α*, *∈*)-RDP dpClassM (**Supplementary Figures S1A–C**). We used the prediction performance (average accuracy and AUC of 10-fold cross-validation) of dpClassM to tune the hyperparameters of dpAE. Next, we built our dpAE with the best-found hyperparameters to mine (*α*, *∈*)-RDP representations for the patients in PD_1_. Finally, we used these 2000-size DP representations of PD_1_ patients to build our final dpClassM model.

**Figure 3:**
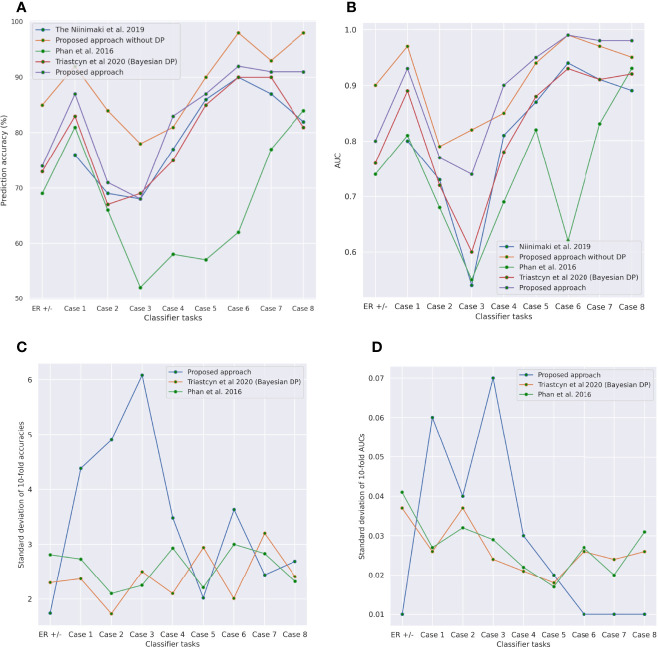
Comparison of prediction performances between our proposed Rényi differential private binary (ER+/- or cancer types) classifiers and baseline models on the METABRIC (34) and the TCGA (33) datasets when privacy budget is 1.0. **(A, B)** Comparison of binary (ER+/- and cancer types) classifiers in terms of mean accuracy (%) and mean AUC, respectively, from the 10-fold cross-validation **(C, D)** Comparison of standard deviations of accuracy (%) and AUC, respectively, from the 10-fold cross-validation.

For the TCGA dataset, **Figures 3A, B** shows the comparison (mean accuracy and AUC of 10-fold cross-validation) of dpClassM against the baselines (25, 31, 32) with the same privacy budget (*∈* = 1.0). In **Figures 3A, B**, the x-axis represents the ER status (ER+/-) and eight pairs of cancer types (number cases of the **Supplementary Table S1**) that we choose to perform our experiments. **Figures 3C, D** shows the standard deviations of accuracies and AUCs from 10-fold cross-validation. **Figures 3A, B** clearly shows that our proposed dpClassM has significant improvement for the ER+/- and each of the eight cancer types prediction performances (accuracy and AUC) then the baselines. Similar to the baselines, the prediction performance of dpClassM for the TCGA cases also varies for different cases because of two reasons: variation in the total number of samples and the imbalance distribution of samples in the two classes (i.e., cancer type pairs). Intuitively, our proposed dpClassM has lesser prediction performance than its non-private version because of the external noise we added during the training of dpClassM (**Figures 3A, B**). Our dpClassM did not learn the actual weight parameters; instead, we used perturbed weight parameters to build dpClassM. The **Supplementary Table S2** also compares the prediction performances among the proposed framework and baselines for five different privacy budgets. Our proposed framework achieved improved prediction performance in all cases in terms of accuracy and AUC. The above comparison indicates our proposed framework’s superiority under a stricter privacy budget than the baselines. We have also added **Supplementary Table S4** in our supplementary with the prediction performance in terms of 95% confidence intervals [similar to (42)] for accuracy (%) and AUC for the METBRIC dataset and the numbered cases from the **Supplementary Table S1**.

### 3.2 Differential Private Regression Analysis

We trained linear regression models (dpRegMs) for each of these 265 drugs from the GDSC dataset to predict their sensitivity in cell lines. This dataset contains lots of missing values because all 265 drugs were not tested in all the ~ 1,000 cell lines. For each of the 265 linear regression models, we only kept the samples (i.e., cell lines) for which that drug was tested. Therefore, the total number of samples in each linear regression model varies from ~350 to ~ 850. Intuitively, if we build dpRegM using these small numbers of high-dimensional gene expression samples, then dpRegM will become prone to the overfitting problem. Hence, we first built a dpAE to extract a 2000-size low-dimensional representation for each of the samples in GDSC. If we build dpRegM on these low-dimensional representations, it is less likely for dpRegM to overfit the training data because of the fewer parameters to be learned by dpRegM. We considered the modified METABRIC dataset as the second private dataset. Then we followed the approach of Section 3.1 to train a hyperparameter tuned dpAE using the modified METABRIC dataset. This trained dpAE was used to extract 2000-size representations for every sample in the GDSC. Finally, we used these low-dimensional representations of the GDSC to build the (*α*, *∈*)- RDP regression model (dpRegM) to predict the drug sensitivity for each of the 265 drugs in cancer cell lines.

In **Table 1**, we showed the performance of our proposed framework for drug sensitivity prediction. We used Spearman’s rank correlation coefficient (43) to evaluate the performance of our differential private linear regression models. Spearman’s rank correlation coefficient (SRCC) measures the correlation between the predicted ranking of the cell lines and the cell lines’ original ranking. SRCC values can be between -1 (perfect negative correlation between the predicted vs. original labels) to 1 (perfect positive correlation between the predicted vs. original labels). We have built 265 differential private drug sensitivity linear regressors. We used 10-fold cross-validation to measure the performance of each of the linear regression models. We considered the mean of SRCCs as the final prediction performance of (*α*, *∈*)- RDP dpRegM.

**Table 1:** Comparison of drug sensitivity prediction performance in terms of average Spearman’s rank correlation coefficients of differential private and non-private models.

Framework	Dataset for representation learning	Privacy Status	Spearman’srank correlation coefficient
(25)	Redistributed TCGA	Private	0.25
(8)	None	Private	0.18
		Non-private	0.26
Bayesian DP (31)	None	Private	0.20(STD 0.057)
**Proposed framework**	METABRIC	Private	0.20(STD 0.051)
		Non-private	0.22(STD 0.043)
	TCGA(Original)	Private	0.26(STD 0.045)
		Non-private	0.28(STD 0.044)

The privacy budget was ∈ = 1.0 for all differential private models. The “Proposed framework” means the differential private model, and the STD represents the standard deviation.

**Table 1** shows the averaged SRCC from all 265 dpRegM models. We can see that our proposed framework’s SRCC in predicting drug sensitivity is higher than the baselines (8, 25) when we used the TCGA dataset to build our data representation learning model (dpAE). However, we have a slightly smaller SRCC than the (25), when we used METABIRC to build dpAE. Nevertheless, this was expected as METABIRC contains discrete values (0,1 and -1), and TCGA contains continuous values (similar to GDSC). Therefore, dpAE, which was trained on TCGA, extracted a more similar representation of GDSC, which leads to the best drug sensitivity prediction performance.

In addition, we also used TCGA dataset to build dpAE, which extracts low-dimensional private deep representation from the GDSC dataset. Then, we used the dpAE extracted low-dimensional GDSC dataset to build our proposed dpRegM for each of the drugs from the GDSC dataset (**Supplementary Figure S1 **and** Supplementary Algorithm S1**). In (25), the TCGA dataset was redistributed to match the GDSC dataset distribution to build a data representation learning model. Such data redistribution is a blatant privacy violation, which was also indicated by the authors. In such cases, an adversary may analyze the public data to extract private information from the private dataset. However, the baseline (25) achieved ~ 0.25 averaged SRCC. Unlike the baseline approach, dpRegM without dataset distribution overcomes the privacy risks from the dataset redistribution procedure, yet dpReGM outperforms (i.e., ~0.27 averaged SRCC) the baseline approaches. **Table 1** also shows the averaged SRCC of all 265 non-private versions of dpRegMs (i.e., without inserting any external noise to the weight parameters during the training phase). Intuitively, all non-private version models of the proposed framework have improved the averaged Spearman’s rank correlation coefficient than their corresponding differential private versions.

Of note, each of our DL models (dpAE, dpClassM, and dpRegM) in the proposed framework are independent of one another. At first, we build Rényi Differential Privacy (RDP) incorporated dpAE model with privacy budget 1.0. RDP used an account function to keep track of the privacy budget spent during each batch-wise model training. Then, we used dpAE representations with another privacy budget 1.0 to build classifiers and regressors. We have added training details of dpAE, dpClassM and dpRegM in the **Supplementary File**. In addition, we have added the dpClassM’s performance for the eight cases (**Supplementary Table S1**) for five different privacy budgets in the **Supplementary Table S2**. Besides, **Supplementary Table S3** shows the list of hyperparameters that were used to build differential private models. List of hyperparmeters that were tested during the training of our DL models (dpAE, dpClassM, and dpRegM) can be found in **Supplementary Table S5–S9**. Finally, the comparison of hyperparameters (during the representation learning) with the previous state-of-the art is shown in **Supplementary Table S10**.

### 3.3 Significance of the Proposed Framework

Our proposed framework incorporated a state-of-the-art differential privacy mechanism in two different stages: low-dimensional feature extraction and binary classification or linear regression. We used (*α*, *∈*)-RDP mechanism to build DP models which has higher utility than the *∈*-DP (10) models because the (*α*, *∈*)-RDP mechanism allows additional leakage of information. However, we used this privacy mechanism on the noisy data to build dpAE. Later, we used the extracted features of dpAE to build dpClassM and dpRegM. Therefore, we have mitigated private data leakage by the (*α*, *∈*)- RDP model because dpClassM and dpRegM did not use the original patient’s private data. An adversary with strong background knowledge can only access (*α*, *∈*)- RDP perturbed data to induce private information from the dataset. Hence, such an adversary can only infer perturbed (by a factor of *∈*) private information rather than accurate private information. In short, all of our models can preserve patients’ sensitive private information up to a factor of *∈*.

In our experiments for building differential private (*∈* 1.0) classifiers and regressors, first we extracted low-dimensional private (*∈* 1.0) dpAE representations from the original raw data. Then, we used these differential private (*∈* 1.0) representations to build our classifiers and regressors using 10-fold cross-validation. Finally, we used the prediction performances of our classifiers and regressors from these 10-folds to tune their hyperparameters. This tuning approach is not violating any privacy as we are not publishing any data (rather we publish the final model only) and our models were tuned based on the differential private (*∈* 1.0) representation instead of the original raw data.

Our proposed models, dpClassM and dpRegM showed better prediction performances in breast cancer status and TCGA’s cancer type classification; and GDSC’s drug sensitivity prediction, respectively, than baseline approaches (25, 32). We believe that this performance gain came from using the deep learning based methods and our choice of differential privacy algorithm in our framework. We know from the previously published literature that DL based approaches are usually more suitable to analyze high-dimensional gene expression data in terms of prediction performance than traditional machine learning methods. Besides, we incorporated the dropout technique into our framework. Dropout improves the generalization ability of our model towards the unseen data than the baseline models. Besides, unlike baseline models, our DL models are non-linear. Usually, it is more difficult to find a distinct trainable pattern in a linear space, to perform complex optimization problems such as classification or linear regression than in a non-linear space. In addition, Rényi differential privacy is more suitable for real-life applications than *∈*-DP.

Our proposed framework (Algorithm 1) neither publishes the dpAE representation nor the dense layer representations of dpClassM and dpRegM. Instead, the proposed framework publishes only the trained (*α*,*ilon*)-RDP models (dpAE, dpClassM, and dpRegM). Similar to the works of Abadi et al. (12), first, we used the *ℓ*_2_ norm gradient clipping during the stochastic gradient descent (SGD) process. This step allows us to control the sensitivity of any single input data on the gradients. Then, we used the (*α*, *∈*)-RDP approach to perturb the gradients. Finally, these perturbed gradients update model (dpAE, dpClassM and dpRegM) parameters (i.e., weights). (*α*, *∈*)-RDP ensures that each step of the SGD is differentially private. Thus, the final output model achieves a certain level of differential privacy under the composition property (30, 44). Therefore, our final trained models (dpAE, dpClassM, and dpRegM) contain only noisy weight parameters. According to Mironov (30), these noisy weight parameters are (*α*, *∈*)-RDP preserved by post-processing. Consequently, an adversary will not be able to infer any privacy-sensitive training data confidently. Furthermore, Mironov (30) mathematically proved that the output from the adaption sequential composition of two RDP mechanisms preserves the RDP (composition property). Hence, we can say that the proposed framework provides a formal privacy guarantee on the published models (dpAE, dpClassM, and dpRegM). Therefore, the proposed framework of dpAE will not violate any privacy of the training data. Similarly, the published dpClassM and dpRegM will not violate any privacy of the training data. Please be noted that the published dpAE can be treated as a form of the pre-trained model to be used by other researchers to extract a lower-dimensional representation of their local dataset. Then, local data holders may use the extracted representation to perform further analysis (for example, they can build their dpClassM).

Our proposed differential private framework is not limited to use to predict BCS, CT, and drug sensitivity. For example, there are three pharmaceutical companies and each of them has a private library of small molecules. Of note, these companies do not want to share their library. Now, assume that each company wants to build a DL model to predict a candidate from their library, which can be used as a drug. However, none of the three libraries has enough molecules to build a robust DL based drug candidate identification model. In this scenario, each company can take our dpAE to extract the (*α*, *∈*)- RDP representation of their library. Hence, dpAE will allow companies to share their own private data library while preserving their raw libraries’ privacy. This approach will equip each company with a larger number of training samples. Each company can also use our dpClassM (to predict whether a molecule is a candidate to be used as a drug) or dpRegM (to predict a drug candidate’s sensitivity) to build a (*α*, *∈*)- RDP classifier or linear regressor model with better utility. Now, companies can publicly release these models for commercial use. An adversary with strong background knowledge about these companies’ libraries can not precisely infer a small molecule’s original properties from the published (*α*, *∈*)- RDP model. Therefore, the companies’ privacy remains intact to a factor (*∈*). In a nutshell, if someone wants to build a state-of-the-art DL classifier and linear regressor with multiple private datasets or a combination of public and private datasets, then one can choose to work with our framework (dpClassM for classification or dpRegM for linear regression) while maintaining the privacy of the private datasets within a predefined privacy budget *∈*.

## 4 Conclusion

This study predicts breast cancer status, cancer type, and drug sensitivity in cancer cell lines using sensitive human genomic data while preserving individuals’ privacy. We hypothesized that our proposed framework would protect individuals’ privacy of the dataset even if the model trained on this dataset is shared with other organizations, while providing improved utility than the previous state-of-the-art baseline approach. Our experimental results suggest the superiority of our proposed framework in the classification of breast cancer status and cancer type over the baseline. Furthermore, in differential private drug sensitivity prediction, unlike the baseline, the prediction of our proposed framework outperformed previous state-of-the-art baseline results using private datasets only. In brief, the proposed framework achieves improved utility while guaranteeing individuals’ privacy than existing approaches. Of note, we perturbed our model’s parameters to build our differential private model in all experimented tasks (i.e., breast cancer status and cancer type classification, and drug sensitivity prediction). Hence, no adversary can infer with sufficient confidence about the individuals’ original raw input data even if we publish our trained models. This attractive property will allow interested parties (e.g., individuals, hospitals, and pharmaceutical companies) to integrate privacy-sensitive data from multiple sources. Consequently, they can build data-hungry deep learning based models without disclosing any raw privacy-sensitive input data.

Our experiments used a private sparse binary dataset (METABIRC) to extract a low-dimensional representation of a continuous valued data set (GDSC). In the future, we will try to collect and include another such private data set with continuous valued gene expression in the proposed framework. Intuitively, the addition of such a private dataset will produce a more accurate low-dimensional representation of the GDSC. Consequently, the proposed framework will provide improved drug sensitivity prediction performance. Another future work of this study would be the extensive hyperparameter tuning for DL (e.g., number of layers, number of neurons per layer) models. This will likely improve the prediction performance of each DL model.

## Data Availability Statement

Publicly available datasets were analyzed in this study. This data can be found here: Three datasets (TCGA: The Cancer Genome Atlas Program (https://www.cancer.gov/about-nci/organization/ccg/research/structural-genomics/tcga); GDSC: Genomics of Drug Sensitivity in Cancer (https://www.cancerrxgene.org/); METABRIC: Molecular Taxonomy of Breast Cancer International Consortium) (https://ega-archive.org/studies/EGAS00000000083) were analyzed in the study. All of them are publicly available.

## Author Contributions

MI: Conceptualization, Formal analysis, Methodology, Software, Validation, Visualization, Writing - original draft. NM: Supervision, Writing - review editing. YW: Supervision, Funding acquisition, Writing - review editing. PH: Conceptualization, Supervision, Project administration, Resources, Funding acquisition, Writing - review editing. PH is the holder of Manitoba Medical Services Foundation (MMSF) Allen Rouse Basic Science Career Development Research Award. All authors listed have made a substantial, direct, and intellectual contribution to the work and approved it for publication.

## Funding

This work has been supported by the University of Manitoba Graduate Fellowship (UMGF), and The Natural Sciences and Engineering Research Council of Canada (NSERC) Visual and Automated Disease Analytics (VADA) program. PH is the holder of Manitoba Medical Services Foundation (MMSF) Allen Rouse Basic Science Career Development Research Award.

## Conflict of Interest

The authors declare that the research was conducted in the absence of any commercial or financial relationships that could be construed as a potential conflict of interest.

## Publisher’s Note

All claims expressed in this article are solely those of the authors and do not necessarily represent those of their affiliated organizations, or those of the publisher, the editors and the reviewers. Any product that may be evaluated in this article, or claim that may be made by its manufacturer, is not guaranteed or endorsed by the publisher.
