# Supplementary material for: Differential Private Deep Learning Models for Analyzing Breast Cancer Omics Data
Source: Front Oncol. 2022 Jun 23;12:879607. doi: 10.3389/fonc.2022.879607 (PMC9259987; doi:10.3389/fonc.2022.879607)
Supplement: Supplementary file 1 [file DataSheet_1.docx]

Supplementary Material

In the method proposed in this investigation, first, we built (α, ε)- Rényi differential private (RDP) (Mironov, 2017) deep leaning based stacked denoising autoencoder (SDAE) to learn underlying data representation from a private dataset. Then, we transfer the learning from the SDAE to build deep learning based RDP classifiers to predict a patient's breast cancer status and cancer type. Similarly, we transfer the learning from the SDAE to build deep learning based RDP regressors from another private dataset to predict the sensitivity of drugs.

**Training details of differential privacy models**

**A. Training details of dpAE**

We have trained our proposed dpAE with the following best found hyperparameters: batch size = 32, epochs = 15, l2 norm clip = 1, noise multiplier = 1.121 and learning rate = 0.0001. First, dpAE receives a vector of size 14,100 (when using the CNA dataset) or 11,700 (when using the TCGA dataset). This input vector passes through 8000, 4000, 2000 size fully connected dense layers. Here, 8000 and 4000 are two intermediate (hidden) layer dimensions. The latent space dimension is 2000. Then, we decode this 2000-size latent vector to the original size input vector using 4000, 8000 and 14100 (when used CNA dataset) or 11,700 (when used TCGA dataset) size three dense layers. Finally, we measure the reconstruction loss (mean squared error) from the last decoded layer with the noisy version of the input. Then, we perturbed the gradients using RDP. Finally, we update the dpAE parameters with these perturbed gradients. Experiments for different hyperparameters used to train dpAE are shown in **Table S5 and Table S6.**

**B. Training details of dpClassM**

We have trained our proposed dpClassM with the following best found hyperparameters: batch size = 8, epochs = 15, l2 norm clip = 0.5, noise multiplier are mentioned in Table S3, for different privacy budgets and learning rate = 0.0001. First, our proposed dpClassM receives a vector of size 2000. Then dpClassM processes the input vector with two dense layers of size 100. Each of the dense layers was followed by a dropout layer with a probability of 0.5 and a ReLU activation. Finally, we obtain the classification prediction using a 2-size dense layer with a softmax activation function. We used the categorical cross-entropy loss function to calculate the classification loss. The gradients from the classification loss were perturbed before updating the dpClassM's parameters. Experiments for different hyperparameters used to train dpClassM are shown in **Table S7 and Table S8.**

**C. Training details of dpRegM**

We have trained our proposed dpClassM with the following best found hyperparameters: batch size = 4, epochs = 15, l2 norm clip = 0.5, noise multiplier are mentioned in Table S3 for different privacy budgets and learning rate = 0.0001. Similar to the dpClassM, our proposed dpRegM also receives a 2000-size vector as input. Then, we processed the input vector with two dense layers of size 100. The output of each dense layer was followed by a dropout layer with a probability of 0.3 and ReLU activation. Finally, we obtain the regression prediction using a dense layer of 1 size with a ReLU activation function. We used the mean squared error (mse) loss function to calculate regression loss. The gradients from the mse loss were perturbed before updating the dpRegM parameters. Experiments for different hyperparameters used to train dpRegM are shown in **Table S9.**

# Supplementary Figures and Tables

## Supplementary Algorithm

**Algorithm S1:** Differential private deep learning based classification and linear regression framework.

**input:** PD_TCGA_ = private dataset TCGA,

PD_GDSC_ = The private GDSC dataset,

label_class_ = Actual class labels for the samples in PD_TCGA_.

label_drugSensitivity =_ Actual drug sensitivity for the samples in PD_GDSC_.

Here, H = Hyperparameters, I = Indicator, F = Features, L = Labels, P = Performance

**output:** Public release of the dpAE, dpClassM, and dpRegM models.

**initialization:**

dpAE_H_ = {number of layers, learning rate, training epochs}, performance = 0, indicator = 10

**Pseudocode:**

Split PD_TCGA_ 🡪 PD_1_ and PD_2_

**while** I > 0 **do**

Build dpAE: f(PD_2_) 🡪 Low-dimensional PD_2_

dpAE_F_ 🡨 dpAE(PD_1_)

Build dpClassM: f(dpAE_F_ , label_class_) 🡪 cancer types

dpClassM_P_ 🡨[accuracy(dpClassM), AUC(dpClassM)]

**if** dpClassM_P_   $\geq$ P **then**

P 🡨 dpClassM_P_

dpAE_H_ 🡨 update with new settings

I 🡨10

**else**

dpAE_H_ 🡨 update with new settings

I 🡨 I – 1

**end**

**end**

Publish dpAE

**if** *Cancer type classification* **then**

Publish dpClassM

**end**

**if** *Drug sensitivity prediction* **then**

dpAE_F_ 🡨 PD_GDSC_

Build dpRegM: f(dpAE_F_ , label_drugSensitivity_) 🡪 drug sensitivity

Publish dpRegM

**end**

## Supplementary Figures

**Figure S1:** Proposed deep learning based differential private framework to perform classification and linear regression tasks with privacy-sensitive biological data. (A) Pipeline to build an underlying data representation learning model (i.e., dpAE) with private data. (B) DL architecture of dpAE. (C) DL architecture of our proposed differential private classifier (i.e., dpClassM). (D) Pipeline to predict the sensitivity of drugs in cell lines. (E) DL architecture of our proposed differential private linear regressor (i.e., dpRegM).

## Supplementary Tables

**Table S1**: The list of cancer-type pairs ordered in descending order by classification difficulty (Niinimäki, Mikko A Heikkilä, *et al.*, 2019).

| **Case no.** | **First type of cancer** | **Second type of cancer** |
| --- | --- | --- |
| **1** | lung squamous cell  carcinoma | head and neck squamous  cell carcinoma |
| **2** | bladder urothelial  carcinoma | cervical and  endocervical cancer |
| **3** | colon adenocarcinoma | rectum adenocarcinoma |
| **4** | stomach adenocarcinoma | esophageal carcinoma |
| **5** | kidney clear  cell carcinoma | kidney papillary  cell carcinoma |
| **6** | glioblastoma multiforme | sarcoma |
|  | adrenocortical cancer | uveal melanoma |
|  | testicular germ  cell tumor | uterine carcinosarcoma |
|  | lung adenocarcinoma | pancreatic adenocarcinoma |
| **7** | ovarian serous  cystadenocarcinoma | uterine corpus  endometrioid carcinoma |
|  | brain lower  grade glioma | pheochromocytoma  and paraganglioma |
|  | skin cutaneous melanoma | mesothelioma |
|  | liver hepatocellular  carcinoma | kidney chromophobe |
| **8** | breast invasive  carcinoma | prostate adenocarcinoma |
|  | acute myeloid  leukemia | diffuse large  B-cell lymphoma |
|  | thyroid carcinoma | cholangiocarcinoma |

**Table S2:** Differential private classification prediction performance in terms of accuracy and AUC for the eight different cases (the numbered cases are from Table S1) of the TCGA dataset. Each case was investigated for five different privacy budgets. The number in parentheses represents the standard deviation of the 10-fold cross-validation.

| **Cancer types**  **(Case no. from Table S1)** | **Methods** | **Metrics** | **Privacy Budget** | | | | |
| --- | --- | --- | --- | --- | --- | --- | --- |
|  |  |  | **0.5** | **0.7** | **1.0** | **1.5** | **2.0** |
| 1 | (Niinimäki, Mikko A Heikkilä, *et al.*, 2019) | Accuracy | 61 | 66 | 76 | 78 | 78 |
|  |  | AUC | 0.62 | 0.68 | 0.77 | 0.80 | 0.83 |
|  | (Phan *et al.*, 2016) | Accuracy | 83 | 79 | 81 | 84 | 85 |
|  |  | AUC | 0.87 | 0.82 | 0.81 | 88 | 90 |
|  | Bayesian DP (Triastcyn and Faltings, 2020) | Accuracy | 95  ( 2.8) | 93  ( 2.8) | 94  (3.46) | 95  (2.19) | 95  ( 2.72) |
|  |  | AUC | 0.96  ( 0.04) | 0.97  (0.04) | 0.94  (0.06) | 0.96  (0.03) | 0.94  (0.04) |
|  | **Proposed framework** | Accuracy | 86  (4.3) | 85  (4.9) | 87  (4.38) | 87  (5.54) | 90  ( 3.1) |
|  |  | AUC | 0.93  (0.03) | 0.91  (0.04) | 0.93  (0.03) | 0.95  (0.03) | 0.96  ( 0.01) |
| 2 | (Niinimäki, Mikko A Heikkilä, *et al.*, 2019) | Accuracy | 59 | 64 | 66 | 71 | 81 |
|  |  | AUC | 0.61 | 0.65 | 0.70 | 0.74 | 0.85 |
|  | (Phan *et al.*, 2016) | Accuracy | 56 | 67 | 66 | 63 | 66 |
|  |  | AUC | 0.52 | 0.63 | 0.68 | 0.69 | 0.75 |
|  | Bayesian DP (Triastcyn and Faltings, 2020) | Accuracy | 65  ( 3.78) | 65  (3.78) | 67  (5.8) | 68  (4.02) | 66  (3.91) |
|  |  | AUC | 0.56  ( 0.06) | 0.56  (0.06) | 0.6  (0.07) | 0.62  (0.09) | 0.57  ( 0.05) |
|  | **Proposed framework** | Accuracy | 66  (3.7) | 64  (6.1) | 71  (4.9) | 75  (3.03) | 75  (3.03) |
|  |  | AUC | 0.72  ( 0.04) | 0.69  (0.08) | 0.77  (0.04) | 0.84  (0.03) | 0.84  (0.03) |
| 3 | (Niinimäki, Mikko A Heikkilä, *et al.*, 2019) | Accuracy | 62 | 66 | 68 | 68 | 65 |
|  |  | AUC | 0.51 | 0.53 | 0.54 | 0.52 | 0.55 |
|  | (Phan *et al.*, 2016) | Accuracy | 47 | 53 | 52 | 68 | 54 |
|  |  | AUC | 0.51 | 0.51 | 0.55 | 0.56 | 0.56 |
|  | Bayesian DP (Triastcyn and Faltings, 2020) | Accuracy | 68  (4.6) | 69  (4.6) | 69  (2.86) | 69 (4.6) | 71 (2.88) |
|  |  | AUC | 0.57  (0.02) | 0.56  (0.02) | 0.6  (0.1) | 0.52  (0.01) | 0.51  (0.01) |
|  | **Proposed framework** | Accuracy | 62  (7.26) | 61  (3.73) | 68  (6.08) | 65  (5.64) | 63  ( 6.8) |
|  |  | AUC | 0.66  ( 0.07) | 0.63  (0.05) | 0.74  (0.07) | 0.71  (0.07) | 0.69  (0.07) |
| 4 | (Niinimäki, Mikko A Heikkilä, *et al.*, 2019) | Accuracy | 74 | 72 | 81 | 82 | 82 |
|  |  | AUC | 0.76 | 0.73 | 0.83 | 0.84 | 0.85 |
|  | (Phan *et al.*, 2016) | Accuracy | 66 | 59 | 58 | 77 | 77 |
|  |  | AUC | 0.66 | 0.56 | 0.69 | 79 | 0.83 |
|  | Bayesian DP (Triastcyn and Faltings, 2020) | Accuracy | 67  ( 8.2) | 67  ( 8.3) | 70( 2.8) | 69  ( 7.2) | 68  ( 5.4) |
|  |  | AUC | 0.52  (0.01) | 0.53  (0.02) | 0.58  (0.03) | 0.54  (0.02) | 0.55  (0.04) |
|  | **Proposed framework** | Accuracy | 76  ( 4.81) | 80  (4.96) | 83  (3.48) | 85  (4.85) | 85  (4.85) |
|  |  | AUC | 0.85  ( 0.03) | 0.89  (0.03) | 0.9  (0.03) | 0.93  (0.03) | 0.93  (0.03) |
| 5 | (Niinimäki, Mikko A Heikkilä, *et al.*, 2019) | Accuracy | 78 | 81 | 86 | 86 | 87 |
|  |  | AUC | 0.79 | 0.81 | 0.89 | 0.9 | 0.91 |
|  | (Phan *et al.*, 2016) | Accuracy | 74 | 74 | 75 | 78 | 80 |
|  |  | AUC | 0.77 | 0.78 | 0.78 | 0.79 | 0.82 |
|  | Bayesian DP (Triastcyn and Faltings, 2020) | Accuracy | 68  (5.44) | 68  (5.4) | 66  (1.98) | 66  (1.98) | 67  (1.97) |
|  |  | AUC | 0.54  (0.04) | 0.55  (0.04) | 0.52  (0.02) | 0.53  (0.02) | 0.53  (0.02) |
|  | **Proposed framework** | Accuracy | 79  (4.78) | 81  (4.11) | 87  (2.02) | 92  (1.86) | 94  (1.4) |
|  |  | AUC | 0.87  ( 0.05) | .86  (0.04) | 0.95  (0.02) | 0.98  (0.01) | 0.98  ( 0.01) |
| 6 | (Niinimäki, Mikko A Heikkilä, *et al.*, 2019) | Accuracy | 83 | 87 | 90 | 91 | 92 |
|  |  | AUC | 0.85 | 0.88 | 0.92 | 0.92 | 0.92 |
|  | (Phan *et al.*, 2016) | Accuracy | 68  ( 8.5) | 68  ( 8.5) | 69  ( 8.2) | 68  ( 8.5) | 69  ( 8.5) |
|  |  | AUC | 0.6  ( 0.11) | 0.6  ( 0.1) | 0.58  ( 0.1) | 0.61  (0.11) | 0.6  ( 0.11) |
|  | Bayesian DP (Triastcyn and Faltings, 2020) | Accuracy | 76 | 78 | 81 | 84 | 85 |
|  |  | AUC | 0.77 | 0.8 | 0.84 | 0.85 | 0.88 |
|  | **Proposed framework** | Accuracy | 85  ( 4.89) | 88  ( 4.5) | 92  (3.63) | 92  (3.67) | 93  ( 2.7) |
|  |  | AUC | 0.94  ( 0.04) | 0.96  (0.02) | 0.99  (0.01) | 0.98  (0.01) | 0.99  ( 0.01) |
| 7 | (Niinimäki, Mikko A Heikkilä, *et al.*, 2019) | Accuracy | 81 | 83 | 88 | 91 | 91 |
|  |  | AUC | 0.83 | 0.84 | 0.9 | 0.94 | 0.94 |
|  | (Phan *et al.*, 2016) | Accuracy | 79 | 79 | 82 | 83 | 86 |
|  |  | AUC | 0.8 | 0.8 | 0.83 | 0.85 | 0.79 |
|  | Bayesian DP (Triastcyn and Faltings, 2020) | Accuracy | 88  ( 3.3) | 89  (3.37) | 90  (3.95) | 88  (3.37) | 90  (3.95) |
|  |  | AUC | 0.54  ( 0.04) | 0.52  (0.06) | 0.52  (0.02) | 0.52  (0.06) | 0.52  ( 0.06) |
|  | **Proposed framework** | Accuracy | 82  ( 8.07) | 89  (4.15) | 91  (2.44) | 91  (2.43) | 90  (2.8) |
|  |  | AUC | 0.95  (0.04) | 0.99  (0.01) | 0.99  (0.01) | 0.99  (0.01) | 0.99  (0.01) |
| 8 | (Niinimäki, Mikko A Heikkilä, *et al.*, 2019) | Accuracy | 82 | 83 | 83 | 86 | 87 |
|  |  | AUC | 0.8 | 0.8 | 0.84 | 0.88 | 0.9 |
|  | (Phan *et al.*, 2016) | Accuracy | 83 | 87 | 84 | 87 | 89 |
|  |  | AUC | 0.91 | 0.92 | 0.95 | 0.93 | 0.92 |
|  | Bayesian DP (Triastcyn and Faltings, 2020) | Accuracy | 84  (5.36) | 83  (5.36) | 80  (5.23) | 84  (5.36) | 80  (5.23) |
|  |  | AUC | 0.76  ( 0.09) | 0.76  (0.09) | 0.63  (0.12) | 0.76  (0.09) | 0.63  ( 0.9) |
|  | **Proposed framework** | Accuracy | 91  ( 3.67) | 89  ( 5.9) | 91  (3.69) | 93  (1.72) | 97  (3.03) |
|  |  | AUC | 0.97  ( 0.02) | 0.97  (0.03) | 0.98  (0.01) | 0.99  (0.01) | 0.99  ( 0.01) |

**Table S3:** List of the noise multiplier values for different representation learning models and their associated classifiers for the proposed framework. We used batch size = 32, learning rate = 0.001 for the representation learning models and we used batch size = 8 and learning rate = 0.0001 for the classifiers. The case numbers represent the types of cancer in Table S1.

| **Dataset** | **Privacy budget** | **Representation learning model (dpAE)** | **Classification model (dpClassM)** |
| --- | --- | --- | --- |
| TCGA case 1 | 0.5 | 2.79 | 2.41 |
|  | 0.7 | 2.78 | 1.79 |
|  | 1.0 | 1.54 | 1.35 |
|  | 1.5 | 1.165 | 1.091 |
|  | 2.0 | 0.998 | 0.95 |
| TCGA case 2 | 0.5 | 2.3 | 3.32 |
|  | 0.7 | 1.73 | 2.44 |
|  | 1.0 | 1.35 | 1.8 |
|  | 1.5 | 1.07 | 1.32 |
|  | 2.0 | 0.933 | 1.105 |
| TCGA case 3 | 0.5 | 2.292 | 3.1 |
|  | 0.7 | 1.71 | 2.27 |
|  | 1.0 | 1.335 | 1.51 |
|  | 1.5 | 1.065 | 1.25 |
|  | 2.0 | 0.931 | 1.058 |
| TCGA case 4 | 0.5 | 2.299 | 2.83 |
|  | 0.7 | 1.72 | 2.1 |
|  | 1.0 | 1.345 | 1.5525 |
|  | 1.5 | 1.07 | 1.175 |
|  | 2.0 | 0.902 | 1.003 |
| TCGA case 5 | 0.5 | 2.33 | 2.17 |
|  | 0.7 | 1.737 | 1.64 |
|  | 1.0 | 1.355 | 1.285 |
|  | 1.5 | 1.075 | 1.028 |
|  | 2.0 | 0.937 | 0.9 |
| TCGA case 6 | 0.5 | 2.2655 | 3.1 |
|  | 0.7 | 1.71 | 1.68 |
|  | 1.0 | 1.3355 | 1.245 |
|  | 1.5 | 1.065 | 1.056 |
|  | 2.0 | 0.929 | 1.367 |
| TCGA case 7 | 0.5 | 1.925 | 2.95 |
|  | 0.7 | 1.531 | 1.61 |
|  | 1.0 | 1.245 | 1.211 |
|  | 1.5 | 1.011 | 1.7 |
|  | 2.0 | 0.9 | 1.03 |
| TCGA case 8 | 0.5 | 2.43 | 2.45 |
|  | 0.7 | 1.82 | 1.83 |
|  | 1.0 | 1.28 | 1.38 |
|  | 1.5 | 1.103 | 1.08 |
|  | 2.0 | 0.955 | 0.94 |
| METABRIC | 0.5 | 5.7 | 2.92 |
|  | 0.7 | 4.2 | 2.17 |
|  | 1.0 | 3 | 1.62 |
|  | 1.5 | 2.13 | 1.245 |
|  | 2.0 | 1.7 | 1.066 |

**Table S4: Comparison of classification performances in terms of 95% confidence intervals (lower bound – upper bound) for accuracy (%) and AUC for the METBRIC dataset and the numbered cases are from Table S1.**

| **Classification problem** | **Frameworks** | **Accuracy** | **AUC** |
| --- | --- | --- | --- |
| ER +/-  (**METBRIC**) | (Phan *et al.*, 2016) | 67.7-71.1 | 0.724-0.771 |
|  | Bayesian DP (Triastcyn and Faltings, 2020) | 69.2-75.3 | 0.744-0.789 |
|  | **Proposed framework** | 74.2-79.6 | 0.781-0.828 |
| Case 1 | (Niinimäki, Mikko A Heikkilä, *et al.*, 2019) | 74.3-79.4 | 0.751-0.802 |
|  | (Phan *et al.*, 2016) | 78.2-84.1 | 0.788-0.833 |
|  | Bayesian DP (Triastcyn and Faltings, 2020) | 90.2-97.4 | 0.872-0.973 |
|  | **Proposed framework** | 83.1-91.3 | 0.898-0.964 |
| Case 2 | (Niinimäki, Mikko A Heikkilä, *et al.*, 2019) | 63.6-69.1 | 0.678-0.744 |
|  | (Phan *et al.*, 2016) | 58.2-72.1 | 0.612-0.722 |
|  | Bayesian DP (Triastcyn and Faltings, 2020) | 61.4-72.2 | 0.523-0.672 |
|  | **Proposed framework** | 68.1-73.3 | 0.724-0.813 |
| Case 3 | (Niinimäki, Mikko A Heikkilä, *et al.*, 2019) | 65.2-70.1 | 0.51-0.577 |
|  | (Phan *et al.*, 2016) | 48.9-55.6 | 0.511-0.613 |
|  | Bayesian DP (Triastcyn and Faltings, 2020) | 65.7-72.1 | 0.51-0.722 |
|  | **Proposed framework** | 61.9-75.3 | 0.677-0.813 |
| Case 4 | (Niinimäki, Mikko A Heikkilä, *et al.*, 2019) | 78.4-84.3 | 79.2-87.7 |
|  | (Phan *et al.*, 2016) | 54.6-61.2 | 0.665-0.722 |
|  | Bayesian DP (Triastcyn and Faltings, 2020) | 67.1-73.5 | 0.533-0.621 |
|  | **Proposed framework** | 79.9-87.2 | 0.862-93.6 |
| Case 5 | (Niinimäki, Mikko A Heikkilä, *et al.*, 2019) | 84.2-88.1 | 0.868-0.932 |
|  | (Phan *et al.*, 2016) | 74.3-79.8 | 0.751-0.812 |
|  | Bayesian DP (Triastcyn and Faltings, 2020) | 64.9-68.2 | 0.5-0.53 |
|  | **Proposed framework** | 85.3-89.9 | 0.923-0.971 |
| Case 6 | (Niinimäki, Mikko A Heikkilä, *et al.*, 2019) | 87.6-93.1 | 0.889-0.943 |
|  | (Phan *et al.*, 2016) | 60.1-77.8 | 0.53-0.646 |
|  | Bayesian DP (Triastcyn and Faltings, 2020) | 78.8-83.5 | 0.811-0.872 |
|  | **Proposed framework** | 88.9-95.8 | 0.988-1 |
| Case 7 | (Niinimäki, Mikko A Heikkilä, *et al.*, 2019) | 85.6-91.3 | 0.871-93.4 |
|  | (Phan *et al.*, 2016) | 79.1-85.8 | 0.797-0.867 |
|  | Bayesian DP (Triastcyn and Faltings, 2020) | 86.4-94.5 | 0.5-0.544 |
|  | **Proposed framework** | 88.7-94.1 | 0.97-1 |
| Case 8 | (Niinimäki, Mikko A Heikkilä, *et al.*, 2019) | 80.6-86.5 | 0.812-0.876 |
|  | (Phan *et al.*, 2016) | 82.3-87.4 | 0.911-0.98 |
|  | Bayesian DP (Triastcyn and Faltings, 2020) | 73.33-87.2 | 0.5-0.734 |
|  | **Proposed framework** | 87.1-95.2 | 0.956-0.99 |

**Table S5: List of hyperparameters used to train proposed dpAE (data representation learning model) framework. Privacy budget is 1. The best found hyperparameters in terms of cancer type classification accuracy and AUC are in bold. The numbered cases are from the supplementary Table S1**

| **Case Numbers** | **Hyperparameters** | **Hyperparameter values** |
| --- | --- | --- |
| 1 | batch size | [8, 16, **32**] |
|  | epochs | [10, **15**, 20] |
|  | learning rate | [0.1, 0.01, **0.001**] |
|  | dense layer size | 1000, 500, 100 |
|  |  | 200, 1000, 500 |
|  |  | **8000, 4000, 2000** |
| 2 | batch size | [8, 16, **32**] |
|  | epochs | [10, **15**, 20] |
|  | learning rate | [0.1, 0.01, **0.0001**] |
|  | dense layer size | 1000, 500, 100 |
|  |  | 200, 1000, 500 |
|  |  | **8000, 4000, 2000** |
| 3 | batch size | [**8**, 16, 32] |
|  | epochs | [10, **15**, 20] |
|  | learning rate | [0.1, 0.01, **0.001**] |
|  | dense layer size | 1000, 500, 100 |
|  |  | 200, 1000, 500 |
|  |  | **8000, 4000, 2000** |
| 4 | batch size | [**8**, 16, 32] |
|  | epochs | [10, **15**, 20] |
|  | learning rate | [0.1, 0.01, **0.0001**] |
|  | dense layer size | 1000, 500, 100 |
|  |  | 200, 1000, 500 |
|  |  | **8000, 4000, 2000** |
| 5 | batch size | [8, 16, **32**] |
|  | epochs | [10, **15**, 20] |
|  | learning rate | [0.1, 0.01, **0.001**] |
|  | dense layer size | 1000, 500, 100 |
|  |  | 200, 1000, 500 |
|  |  | **8000, 4000, 2000** |
| 6 | batch size | [8, 16, **32**] |
|  | epochs | [10, **15**, 20] |
|  | learning rate | [0.1, 0.01, **0.001**] |
|  | dense layer size | 1000, 500, 100 |
|  |  | 200, 1000, 500 |
|  |  | **8000, 4000, 2000** |
| 7 | batch size | [8, 16, **32**] |
|  | epochs | [10, 15, **20**] |
|  | learning rate | [0.1, 0.01, **0.0001**] |
|  | dense layer size | 1000, 500, 100 |
|  |  | 200, 1000, 500 |
|  |  | **8000, 4000, 2000** |
| 8 | batch size | [8, 16, **32**] |
|  | epochs | [10, **15**, 20] |
|  | learning rate | [0.1, 0.01, **0.0001**] |
|  | dense layer size | 1000, 500, 100 |
|  |  | 200, 1000, 500 |
|  |  | **8000, 4000, 2000** |

**Table S6: List of hyperparameters used to train proposed dpAE (data representation learning model) framework. Privacy budget is 1. The best found hyperparameters in terms of ER+/- cancer classification accuracy and AUC are in bold.**

| **Hyperparameters** | **Hyperparameter values** |
| --- | --- |
| batch size | [8, 16, **32**] |
| epochs | [10, 15, **20**] |
| learning rate | [0.1, 0.01, **0.0001**] |
| dense layer size | 1000, 500, 100 |
|  | 200, 1000, 500 |
|  | **8000, 4000, 2000** |

**Table S7:** **List of hyperparameters used to train proposed dpClassM. Privacy budget is 1. The best found hyperparameters in terms of cancer type classification accuracy and AUC are in bold. The numbered cases are from supplementary Table S1.**

| **Case Numbers** | **Hyperparameters** | **Hyperparameter values** |
| --- | --- | --- |
| 1 | batch size | [**8**, 16, 32] |
|  | epochs | [10, **15**, 20] |
|  | learning rate | [0.1, 0.01, **0.0001**] |
|  | dense layer size | 100, 50 |
|  |  | **100, 100** |
|  |  | 500, 100 |
| 2 | batch size | [**8**, 16, 32] |
|  | epochs | [10, **15**, 20] |
|  | learning rate | [0.1, 0.01, **0.0001**] |
|  | dense layer size | 100, 50 |
|  |  | **100, 100** |
|  |  | 500, 100 |
| 3 | batch size | [**8**, 16, 32] |
|  | epochs | [10, 15, **20**] |
|  | learning rate | [0.1, 0.01, **0.0001**] |
|  | dense layer size | 100, 50 |
|  |  | **100, 100** |
|  |  | 500, 100 |
| 4 | batch size | [**8**, 16, 32] |
|  | epochs | [**10**, 15, 20] |
|  | learning rate | [0.1, 0.01, **0.0001**] |
|  | dense layer size | 100, 50 |
|  |  | **100, 100** |
|  |  | 500, 100 |
| 5 | batch size | [**8**, 16, 32] |
|  | epochs | [10, **15**, 20] |
|  | learning rate | [0.1, 0.01, **0.0001**] |
|  | dense layer size | 100, 50 |
|  |  | **100, 100** |
|  |  | 500, 100 |
| 6 | batch size | [8, **16**, 32] |
|  | epochs | [10, **15**, 20] |
|  | learning rate | [0.1, 0.01, **0.0001**] |
|  | dense layer size | 100, 50 |
|  |  | **100, 100** |
|  |  | 500, 100 |
| 7 | batch size | [**8**, 16, 32] |
|  | epochs | [10, **15**, 20] |
|  | learning rate | [0.1, 0.01, **0.0001**] |
|  | dense layer size | 100, 50 |
|  |  | **100, 100** |
|  |  | 500, 100 |
| 8 | batch size | [**8**, 16, 32] |
|  | epochs | [10, 15, **20**] |
|  | learning rate | [0.1, 0.01, **0.0001**] |
|  | dense layer size | 100, 50 |
|  |  | **100, 100** |
|  |  | 500, 100 |

**Table S8:** **List of hyperparameters used to train proposed dpClassM. Privacy budget is 1. The best found hyperparameters in terms of ER+/- classification accuracy and AUC are in bold.**

| **Hyperparameters** | **Hyperparameter values** |
| --- | --- |
| batch size | [8, 16, **32**] |
| epochs | [10, 15, **20**] |
| learning rate | [0.1, 0.01, **0.0001**] |
| dense layer size | 100, 50 |
|  | **100, 100** |
|  | 500, 100 |

**Table S9:** Comparison of hyperparameters used to train proposed dpRegM. Privacy budget is 1. The best found hyperparameters for drug sensitivity prediction performance in terms of average Spearman’s rank correlation coefficients are bold.

| **Hyperparameters** | **Hyperparameter values** |
| --- | --- |
| batch size | [**4**, 8, 16] |
| epochs | [10, **15**, 20] |
| learning rate | [0.1, 0.01, **0.0001**] |
| dropout probability | [0.5, **0.3,** 0.2] |
| dense layer size | 100, 50 |
|  | **100, 100** |

**Table S10: Comparison of the hyperparameters (during representation learning) of the proposed framework with the hyperparameters of baseline state-of-the-art framework (Niinimäki, Mikko A. Heikkilä, *et al.*, 2019). The numbered cases are from the supplementary Table S1.**

| **Case** | **(Niinimäki, Mikko A. Heikkilä, *et al.*, 2019)** | | | | **Proposed framework** | | | |
| --- | --- | --- | --- | --- | --- | --- | --- | --- |
|  | **Method** | **Log-lr** | **Layers** | **Layer-dim** | **Method** | **SGD-lr** | **Layers** | **Layer-dim** |
| 1 | Variational auto-encoder (VAE) | -3.5 | 1 | 755 | Stacked denoising auto-encoder (SDAE) | 0.001 | 3 | [8000, 4000, 2000] |
| 2 |  | -4.8 | 1 | 1925 |  | 0.0001 | 3 | [8000, 4000, 2000] |
| 3 |  | -5.3 | 2 | 2370 |  | 0.001 | 3 | [8000, 4000, 2000] |
| 4 |  | -5.3 | 2 | 1270 |  | 0.0001 | 3 | [8000, 4000, 2000] |
| 5 |  | -4.3 | 1 | 260 |  | 0.001 | 3 | [8000, 4000, 2000] |
| 6 |  | -4.5 | 1 | 330 |  | 0.001 | 3 | [8000, 4000, 2000] |
| 7 |  | -3.7 | 2 | 1510 |  | 0.0001 | 3 | [8000, 4000, 2000] |
| 8 |  | -3.8 | 1 | 88 |  | 0.0001 | 3 | [8000, 4000, 2000] |
|  |  |  |  |  |  |  |  |  |

**References:**

Mironov, I. (2017) ‘Rényi Differential Privacy’, *Proceedings - IEEE Computer Security Foundations Symposium*. IEEE Computer Society, pp. 263–275. doi: 10.1109/CSF.2017.11.

Niinimäki, T., Heikkilä, Mikko A., *et al.* (2019) ‘Representation transfer for differentially private drug sensitivity prediction’, in *Bioinformatics*. Oxford University Press, pp. i218–i224. doi: 10.1093/bioinformatics/btz373.

Niinimäki, T., Heikkilä, Mikko A, *et al.* (2019) ‘Representation transfer for differentially private drug sensitivity prediction’, *Bioinformatics*. Oxford Academic, 35(14), pp. i218–i224. doi: 10.1093/BIOINFORMATICS/BTZ373.

Phan, N. H. *et al.* (2016) ‘Differential privacy preservation for deep auto-encoders: An application of human behavior prediction’, in *30th AAAI Conference on Artificial Intelligence, AAAI 2016*.

Triastcyn, A. and Faltings, B. (2020) ‘Bayesian Differential Privacy for Machine Learning’, *37th International Conference on Machine Learning, ICML 2020*. International Machine Learning Society (IMLS), PartF16814, pp. 9525–9534. Available at: https://arxiv.org/abs/1901.09697v5 (Accessed: 22 July 2021).
